# Awareness of chemotherapy-induced nausea and vomiting and adherence to guidelines: results of a multinational and multicenter survey, part of the THRIVE program

**DOI:** 10.1007/s00520-026-10460-0

**Published:** 2026-03-17

**Authors:** Ricardo Caponero, Diego Enrico, Flavia Giudice, Jean-Pierre Ayoub, Nathalie Lapointe

**Affiliations:** 1 Hospital Alemão Oswaldo Cruz, São Paulo, Brazil; 2 Alexander Fleming Cancer Institute, Buenos Aires, Argentina; 3 Knight Therapeutics, São Paulo, Brazil; 4 CHUM, Montréal, Canada; 5 Knight Therapeutics, Montréal, Canada

**Keywords:** CINV prophylaxis, Chemotherapy, Nausea, Vomiting, Emetogenic, Guidelines

## Abstract

**Purpose:**

Chemotherapy-induced nausea and vomiting (CINV) is a common adverse effect that clearly benefits from prophylactic management. Awareness of CINV and adherence to CINV management guidelines was assessed under a continuing medical education program involving a personal practice assessment (PPA)—THRIVE (Training to Help Reduce CINV ratEs).

**Methods:**

Forty-six medical oncologists from Canada (*n* = 21), Brazil (*n* = 20), and Argentina (*n* = 5) answered an anonymous survey of their practices during patient consultations. The questionnaire was developed by a group of medical oncologist experts.

**Results:**

The survey included data on 446 patients with multiple cancer types undergoing treatment with highly emetogenic chemotherapy (HEC; 60%) and moderately emetogenic chemotherapy (MEC; 40%). Although 65% of respondents reported using more than one guideline to establish CINV management protocols, discrepancies between respondents’ classifications and major guidelines were observed, particularly for newer agents and carboplatin dosing. In addition, 11% of respondents did not discuss personal additional risk factors for CINV with patients. Regarding CINV prophylactic protocol for MEC, 39% of respondents did not include neurokinin 1 receptor antagonist (NK-1 RA) in the regimen for patients with additional risk factors on MEC. The survey also revealed significant variability in the time points adopted for assessing CINV, with 35% of physicians relying solely on spontaneous reports by patients of delayed CINV.

**Conclusion:**

There is a pressing need to explore and support initiatives for effective implementation of guidelines and identifying the causes of nonadherence.

**Supplementary Information:**

The online version contains supplementary material available at 10.1007/s00520-026-10460-0.

## Introduction

Chemotherapy-induced nausea and vomiting (CINV) is a frequent complication in patients undergoing emetogenic chemotherapy [1], with some agents having a CINV risk of up to 90% [2].

CINV is the most common and troubling side effect patients experience during chemotherapy, negatively impacting both compliance with cancer therapies and quality of life [1]. When left untreated, CINV affects 30–90% of cancer patients and can be associated with premature discontinuation of treatment, reduced quality of life, complications such as dehydration and electrolyte imbalances, and, ultimately, with worse treatment outcomes and increased cost of care [3–7].

Significant advances in CINV treatment and prevention have been made over the past 30 years, including the development of novel antiemetic agents and the use of drug combinations [8]. A range of antiemetic guidelines with regular updates are available, designed to enhance the treatment and prevention of CINV [2, 3, 9, 10]. Good adherence to these guidelines has the potential to substantially reduce the incidence of CINV and the need for specialized healthcare visits and emergency room care [11, 12]. However, emerging evidence shows that the current level of adherence to these guidelines in clinical practice is low [11, 13, 14]. Thus, increasing adherence to these guidelines can lead to significant improvements in CINV management and patient outcomes.

The THRIVE (Training to Help Reduce CINV ratEs) program was developed to assess how cancer patients on moderately emetogenic chemotherapy (MEC) or highly emetogenic chemotherapy (HEC) are managed for CINV prophylaxis and to what extent the practices of healthcare professionals (HCPs) comply with current international guidelines for the prevention of CINV. This report presents the results of a multicenter survey assessing HCP awareness of and adherence to CINV guidelines within the THRIVE program.

## Methods

### Design and setting

This was a multicenter survey conducted in Canada, Brazil, and Argentina between March and December 2023. The survey was designed to assess HCPs’ knowledge and adherence to evidence-based guidelines for the prevention and management of CINV. Four medical oncology experts—two from Canada, one from Brazil, and one from Argentina—developed the questionnaire as part of the *Personal Practice Assessment *(PPA), a continuing medical education program based on international recommendations for CINV management [2, 3].

### Participants

Eligible participants were licensed clinical oncologists actively treating adult cancer patients with chemotherapy. Physicians were randomly selected through a computer-generated randomization process from national oncology registries and professional networks. Invitations were distributed electronically, and participants accessed the survey via a secure online link. All responses were collected anonymously during regular clinical practice hours.

### Data collection and questionnaire

The structured questionnaire included two main sections. The first explored oncologists’ clinical practices regarding prophylaxis and management of CINV, while the second collected data on patient demographics and treatment characteristics. The questionnaire comprised two parts: part one, each HCP completed a one-time section reporting on their own clinical practice; part two, each participating oncologist provided data for ten adult patients who had recently received, or were scheduled to receive, a HEC or MEC regimen. After completing the initial HCP section, respondents retained access to the online platform throughout the survey period (from March to December 2023), allowing them to enter data for the patient-specific section each time they conducted an eligible consultation. This approach ensured that patient information was recorded contemporaneously, minimizing recall bias and enhancing data reliability. The complete survey instrument is available in the Supplementary Material.

### Data handling and ethical considerations

All data were anonymized at the point of collection, and no patient identifiers were recorded. Participation was voluntary, and completion of the questionnaire implied consent to use the aggregated data for research and educational purposes.

### Statistical analysis

Data were compiled in Microsoft Excel for descriptive analysis. Categorical variables were summarized using frequencies and percentages, and continuous variables were described with means, standard deviations, and ranges. Subgroup analyses were performed to compare responses among participating countries and between the HEC and MEC treatment groups. Data consistency and completeness were verified prior to analysis.

## Results

### Survey population

All invited physicians answered the survey (*n* = 46). The sample comprised 21 oncologists from Canada, 20 from Brazil, and 5 from Argentina, who collectively provided data on 446 patients receiving HEC or MEC treatment. Table 1 summarizes the characteristics of participating HCP, including years of clinical practice, type of treatment center, and cancer types managed, overall and stratified by country (Canada, Brazil, and Argentina). Table 2 describes the demographic and clinical profile of included patients, encompassing age distribution, performance status, cancer characteristics, treatment context, and emetic risk classification, for the overall cohort and by country. Patients had a wide age range (18 to  ≥ 80 years), two-thirds were female, and most (86%) were in relatively good health (ECOG 0–1).

**Table 1:** Characteristics of healthcare professional practice (percentage)

Characteristics (%)	Total population (*n* = 46)	Canada (*n* = 21)	Brazil (*n* = 20)	Argentina (*n* = 5)
Years practicing				
< 5 years	2	5	5	0
5–15 years	60	43	65	100
> 15 years	38	52	30	0
Types of center				
Academic or university affiliated hospital or cancer center	37	52	20	40
Private hospital or treatment center	51	0	100	80
Public community hospital	26	48	5	20
Community cancer center	2	5	0	0
Types of cancer treated				
Breast	85	90	80	80
Gastrointestinal	76	71	80	80
Genitourinary	67	71	70	60
Gynecologic	63	71	70	60
Thoracic	61	67	65	40
Head and neck	54	57	60	40
Skin	54	57	60	40
Hematologic	48	43	55	40
Endocrine and neuroendocrine	46	38	50	20
Neurologic	35	19	45	20
Musculoskeletal	28	10	40	0
Ocular	9	10	10	0
Other	11	5	20	0

**Table 2:** Characteristics of patients

Age (years, *n* in %)				
	Total population (*n* = 446)	Canada (*n* = 194)	Brazil (*n* = 201)	Argentina (*n* = 51)
18–29	6	5	7	14
30–39	15	14	18	10
40–49	17	17	19	12
50–59	19	23	17	27
60–69	20	27	17	18
70–79	19	11	17	19
≥ 80	4	3	5	0
Gender (%)				
Female	64	70	58	63
Male	36	30	42	37
Performance status (ECOG PS) (%)				
0	48	46	43	59
1	38	39	40	37
2	12	12	14	4
3	2	3	2	0
4	< 1	0	< 1	0
Type of coverage (%)				
Private	63	32	98	74
Public	32	65	2	18
Other	5	3	0	8
Type of cancer (%)				
Breast	39	57	36	37
Gastrointestinal	21	17	23	10
Genitourinary	8	4	12	8
Hematologic	8	9	10	0
Gynecologic	7	3	7	14
Thoracic	6	7	3	0
Head and neck	4	0	4	17
Musculoskeletal	2	0	2	8
Neurologic	2	< 1	0	6
Skin	2	< 1	1	0
Endocrine and neuroendocrine	< 1	0	1	0
Ocular	< 1	0	< 1	0
Other	2	< 1	< 1	0
Cancer staging (%)				
I	4	1	7	6
II	23	35	23	22
III	28	34	25	33
IV	41	27	40	37
Not applicable	4	3	5	2
Cancer treatment (%)				
Neoadjuvant	26	24	23	29
Adjuvant	24	21	23	26
Maintenance	2	0	5	0
Metastatic	42	52	43	31
Not applicable	6	3	6	14
Emetic risk (%)				
High	59	54	64	78
Moderate	41	46	36	22

### Guidelines for CINV management

Sixty-five percent of HCPs reported using more than one guideline to establish protocols for CINV management. Guideline use was as follows: American Society of Clinical Oncology (ASCO) (52%); National Comprehensive Cancer Network (NCCN) (50%); Multinational Association of Supportive Care in Cancer/European Society of Medical Oncology (MASCC/ESMO) (33%); Cancer Care Ontario (CCO) (28%); other (24%); and product labeling (7%). Other included Groupe d'étude en Oncologie du Québec (GEOQ), Agência Nacional de Saúde (ANS), British Columbia (BC) Cancer, Centro de Pesquisas Oncológicas (CEPON), Leaflet/pharmaceutical knowledge, and local protocol.

### Emetic risk according to drug and patient individual factors

Table 3 presents the emetic risk classification of commonly used anticancer agents, comparing respondents’ assessments with classifications from ASCO, MASCC/ESMO, and NCCN guidelines (from 2023). These drugs were selected because, according to expert opinion, they represent the most frequently used chemotherapeutic agents in clinical practice. When comparing respondents’ classifications with major international guidelines (ASCO, MASCC/ESMO, and NCCN), overall concordance was high for well-established regimens such as cisplatin and anthracycline/cyclophosphamide combinations, which were consistently identified as highly emetogenic. Discrepancies were particularly evident for moderately emetogenic agents with evolving or less clearly established classifications, including sacituzumab govitecan and trastuzumab deruxtecan.

**Table 3:** Emetic risk classification of commonly used agents

	Respondents	ASCO	MASCC/ESMO	NCCN
Anthracycline/cyclophosphamide combination	95% high; 5% moderate	High	High	High
Carboplatin AUC < 4	11% high; 89% moderate	Moderate (does not differentiate based on dose)	Moderate (does not differentiate based on dose)	Moderate
Carboplatin AUC ≥ 4	61% high; 39% moderate			High
Cisplatin	89% high; 11% moderate	High	High	High
Sacituzumab govitecan	72% high; 28% moderate	-	Moderate	High
Trastuzumab deruxtecan	68% high; 32% moderate	Moderate	Moderate	High

*ASCO* American Society of Clinical Oncology, *AUC* area under the curve, *MASCC/ESMO* Multinational Association of Supportive Care in Cancer/European Society of Medical Oncology, *NCCN* National Comprehensive Cancer Network

**Fig. 1:**
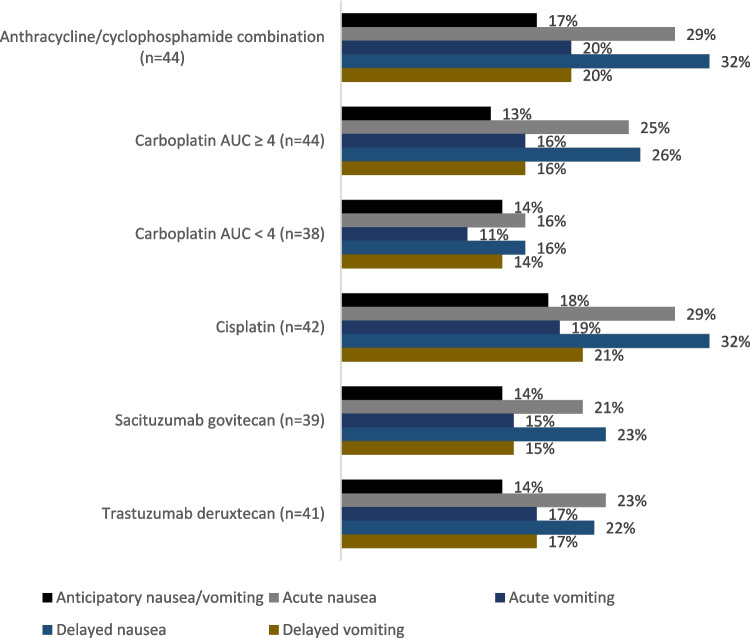
Estimated proportion of patients experiencing CINV while on antiemetic prevention, for commonly used anticancer agents

As shown in Fig. 1, a substantial proportion of patients experienced anticipatory, acute, or delayed CINV despite antiemetic prophylaxis across all commonly used anticancer agents. Respondents stated that 20–30% of their patients on HEC and prophylactic antiemetic regimens experienced acute nausea, with a similar proportion experiencing delayed nausea, while 7–11% experienced anticipatory nausea/vomiting.

**Fig. 2:**
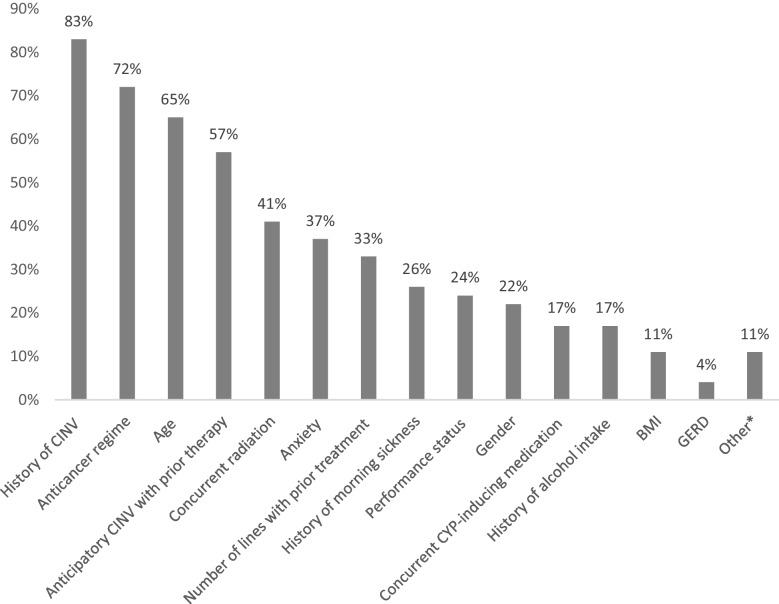
Evaluation of additional risk factors of patients under MEC according to participants for introducing NK-1 RA. *Others include motion sickness, drug coverage (i.e., private insurance), pre-established refractoriness to prophylaxis for MEC, previous major abdominal surgery, and vascular access. BMI: body mass index; CYP: cytochrome P450; GERD: gastroesophageal reflux disease; MEC: moderate emetogenic chemotherapy; NK-1 RA: neurokinin-1 receptor antagonist

**Fig. 3:**
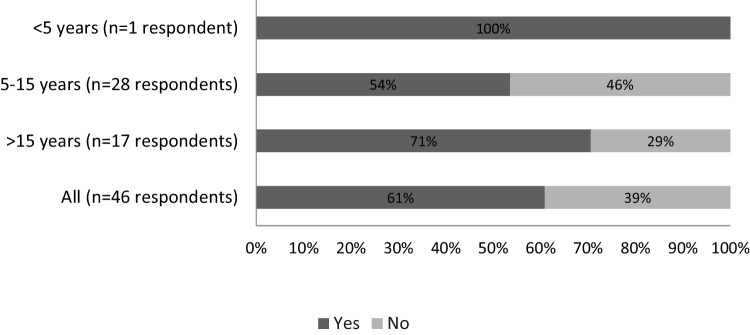
Number of years practicing vs. NK-1 RA use in patients with additional risk factors under treatment with MEC

When evaluating patients on carboplatin and antibody–drug conjugates (ADCs), considered as MEC, 54% of participants failed to consider individual risk factors for CINV. Among respondents not assessing these factors, there was a failure to consider all risk factors. Table 4 summarizes the percentage of respondents who reported considering additional patient-specific risk factors for CINV when managing patients receiving MEC. When deciding on introducing a neurokinin 1 receptor antagonist (NK-1 RA) for patients receiving MEC, over 50% took into account prior therapy, anticancer regimen, age, and anticipatory nausea. Figure 2 summarizes the patient-related risk factors considered by respondents when deciding to introduce an NK-1 RA in patients receiving MEC. Other risk factors were considered by fewer than 40% of respondents, and some factors not included in the guidelines were also taken into account. Figure 3 depicts the proportion of respondents reporting use of an NK-1 receptor antagonist in patients with additional risk factors receiving moderately emetogenic chemotherapy (MEC), stratified by years of clinical practice. Number of years of practice was found to influence the use of NK-1 RA in patients with additional risk factors under treatment with MEC (Fig. 3).

**Table 4:** Percentage of respondents considering additional risk factors for CINV in patients under MEC

Risk factors*	Respondents (%) *N* = 46
History of CINV	83
Age (6–50 years)	65
Anxiety	37
Gender (female)	22
Motion sickness	11
Morning sickness during pregnancy	26
Alcohol consumption	17
High pretreatment expectation of CINV	0

*From NCCN guidelines [2]

*CINV* chemotherapy-induced nausea and vomiting, *MEC* moderately emetogenic chemotherapy

### Assessing CINV: by whom and when?

Participants reported using more than one particular approach to assess CINV: 78% stated this was carried out not only by the oncologist in person, but also by a nurse in person (61%), by patients themselves if presenting symptoms (43%), by the pharmacist (22%), by a nurse over the telephone (13%), by the pharmacist over the telephone (11%), or by patients via self-reporting or questionnaire at the next cycle (13%).

The survey results also showed significant variability in the time points adopted for assessing CINV, with 35% of physicians relying solely on patient spontaneous reports of delayed CINV; these physicians/centers did not perform active evaluations during the days of chemotherapy. Also, 35% of respondents assessed the occurrence of CINV for < 3 days after chemotherapy, not covering the minimum duration of effect of HEC agents; 23% assessed for 3–4 days, and 4% assessed for 5–6 days. Some respondents reported assessing CINV after one week (18%), two weeks (13%), and three weeks (17%).

### Challenges managing CINV

Participants identified three key challenges managing CINV: ensuring access to and coverage for antiemetic therapies, identifying patients receiving MEC who are at higher risk for CINV, and promoting adherence to the antiemetic regimen.

Additionally, respondents noted that dealing with delayed nausea and vomiting, as well as selecting an appropriate antiemetic regimen, is a significant challenge in managing CINV.

### Medication for CINV

**Fig. 4:**
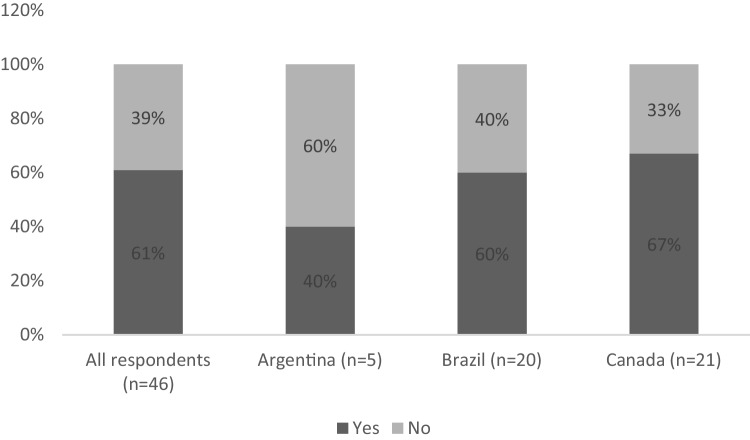
NK-1 RA use in patients with additional risk factors under MEC

Figure 4 shows the proportion of respondents reporting use of an NK-1 receptor antagonist in patients with additional risk factors receiving moderately emetogenic chemotherapy (MEC), overall and by country. Thirty-nine percent of respondents did not use NK-1RA as CINV prophylaxis in patients with additional risk factors under treatment with MEC.

Correlating number of years practicing, 46% of respondents with 5–15 years in practice did not use NK-1 RA as CINV prophylaxis in patients with additional risk factors under treatment with MEC, while this rate was lower in the group of physicians practicing for more than 15 years (29%). Healthcare professionals practicing for under five years were not assessed due to the small sample size (*n* = 1).

## Discussion

Studies indicate that, while adherence to guidelines enhances the management of CINV, the overall adherence rate remains low [16–18]. Notably, adherence to these guidelines is particularly important for patients receiving HEC [19].

In the present assessment, although most respondents reported adhering to multiple guidelines for managing CINV, significant disparities existed between their clinical practices and guideline recommendations. The misclassification rate regarding emetic risk for ADCs and carboplatin (AUC ≥ 4) was high, with respondents often underestimating the emetogenic potential of treatments. Respondents strongly endorsed the effectiveness of antiemetic therapy for managing CINV in HEC, citing that fewer than 30% of patients under antiemetic treatment were likely to experience any CINV symptoms. However, antiemetic prophylaxis for patients on MEC is primarily based on the emetic potential of the treatment regimen, but individual risk factors should be considered for patients under oxaliplatin, as recommended by MASCC/ESMO guideline [2, 3, 10]. Even when individual patient risk factors were considered, clinical practice consistently aligned with only a subset of guideline-recommended factors, while other factors not addressed in the guidelines were also taken into account. According to the NCCN guidelines [2], which address these risk factors more objectively, additional considerations such as younger age, gender, and history of CINV should be taken into account (as listed in Table 4). Customizing antiemetic treatment based on individual risk factors can enhance the effectiveness of antiemetic medications, leading to better management of CINV [20]. It is important to note that guidelines may differ in their classification of emetogenic potential, which could influence how prophylactic recommendations are applied. This variability may partly explain differences in healthcare professionals’ responses and adherence patterns across settings.

The challenges reported by the respondents underscore the complexity of managing CINV and the disparate situations encountered by HCPs among different countries. In Canada, issues such as patient adherence to treatment and identification of high-risk patients are prominent, while access to antiemetic therapy is a significant concern in both Brazil and Argentina. Access and coverage for antiemetic therapy are limited in Brazil and Argentina, as their respective healthcare systems do not provide the same level of care as those in Canada. According to an exploratory descriptive study assessing the availability of antiemetic drugs for the management of CINV in Brazil, agents from all major therapeutic classes are registered in the country; however, drugs such as palonosetron, netupitant + palonosetron, and NK₁ receptor antagonists (aprepitant and fosaprepitant) are not included in the national reimbursement list [15]. This heterogeneity highlights the need for tailored strategies to address these challenges effectively and improve the quality of care for cancer patients undergoing chemotherapy.

Many HCPs did not utilize NK-1 RA for CINV prophylaxis in patients undergoing MEC with additional risk factors. Furthermore, the correlation between the number of years practicing and NK-1 RA usage suggests that more highly experienced practitioners are more likely to use NK-1 RA in these scenarios. This finding highlights the importance of ongoing education and awareness campaigns to promote optimal CINV management practices among HCPs at all experience levels.

This survey provides valuable insights into CINV management across Canada, Brazil, and Argentina. Nonetheless, the survey also reveals persistent challenges in the delivery of care, such as erroneous information and nonadherence to guidelines, regardless of the income level of the country. These findings underscore the need for targeted interventions to address these issues and enhance healthcare quality globally.

This survey presents certain limitations that should be considered when interpreting the findings. The number of HCPs participating in each country was modest, which may have constrained the depth of comparative analyses, although information from more than 400 patients was obtained. Given the voluntary nature of participation, some degree of response bias cannot be ruled out, as those with greater familiarity or interest in CINV management may have been more inclined to respond. The sample may also not fully reflect the diversity of oncology practices within each national context. Moreover, as a descriptive, cross-sectional survey, the results are intended to provide an overview rather than infer causal relationships. Finally, differences in healthcare organization, drug availability, and reimbursement policies among the participating countries should be taken into account when considering the generalizability of the results to other healthcare systems.

Although the present survey was primarily descriptive, its findings highlight opportunities to enhance the implementation of and adherence to CINV management guidelines. The variability observed in antiemetic prescribing practices suggests that greater dissemination of updated international recommendations, coupled with targeted educational initiatives, could improve consistency in clinical decision-making. Integrating guideline-based decision tools into electronic prescribing systems and fostering multidisciplinary discussions on supportive care may also help bridge existing gaps between knowledge and practice. Future research should explore the impact of such interventions on clinical outcomes and patient-reported experiences, ideally through larger and more representative samples across diverse healthcare settings.

## Conclusion

There is a pressing need to explore and support initiatives that implement guidelines for managing CINV effectively, as well as to further investigate the causes of nonadherence. Improving patient outcomes will require focused efforts on education, training, and monitoring of HCPs involved in oncological care.

## Supplementary Information

Below is the link to the electronic supplementary material.

Supplementary file1 (PDF 314 KB)

## Data Availability

Data is provided within the manuscript or supplementary information files.

## Abbreviations

ADCS: Antibody–drug conjugates
ANS: Agência Nacional de Saúde
ASCO: The American Society of Clinical Oncology
BC: British Columbia
CCO: Cancer Care Ontario
CEPON: Centro de Pesquisas Oncológicas
CINV: Chemotherapy-induced nausea and vomiting
GEOQ: Groupe d'étude en Oncologie du Québec
HCP: Healthcare professional
HEC: Highly emetogenic chemotherapy
MASCC/ESMO: Multinational Association of Supportive Care in Cancer/European Society of Medical Oncology
MEC: Moderated emetogenic chemotherapy
NCCN: National Comprehensive Cancer Network
NK-1 RA: Neurokinin 1 receptor antagonist
PPA: Personal practice assessment
THRIVE: Training to help reduce CINV rates
