# Supplementary material for: Awareness of chemotherapy-induced nausea and vomiting and adherence to guidelines: results of a multinational and multicenter survey, part of the THRIVE program
Source: Support Care Cancer. 2026 Mar 17;34(4):334. doi: 10.1007/s00520-026-10460-0 (PMC12995934; doi:10.1007/s00520-026-10460-0)
Supplement: Supplementary file 1 — Supplementary file1 (PDF 314 KB) [file 520_2026_10460_MOESM1_ESM.pdf]

**PRELIMINARY QUESTIONNAIRE (to be answered only once)**

1. In which country and federative unit, province, or territory do you practice?
    - Argentina
        - Autonomous City of Buenos Aires
        - Buenos Aires
        - Catamarca
        - Chaco
        - Chubut
        - Córdoba
        - Corrientes
        - Entre Rios
        - Formosa
        - Jujuy
        - La Pampa
        - La Rioja
        - Mendoza
        - Misiones
        - Neuquén
        - Río Negro
        - Salta
        - San Juan
        - San Luis
        - Santa Cruz
        - Sante Fe
        - Santiago del Estero
        - Tierra del Fuego, Antártida et Islas del Atlántico Sur
        - Tucumán
    - Brazil
        - Acre
        - Alagoas
        - Amapá
        - Amazonas
        - Bahia
        - Ceará
        - Distrito Federal
        - Espírito Santo
        - Goiás
        - Maranhão
        - Mato Grosso
        - Mato Grosso do Sul
        - Minas Gerais
        - Pará
        - Paraíba
        - Paraná
        - Pernambuco
        - Piauí

   - Rio de Janeiro
   - Rio Grande do Norte
   - Rio Grande do Sul
   - Rondônia
   - Roraima
   - Santa Catarina
   - São Paulo
   - Sergipe
   - Tocantins
- ☐ Canada
   - Alberta
   - British Columbia
   - Manitoba
   - New Brunswick
   - Newfoundland and Labrador
   - Northwest Territories
   - Nova Scotia
   - Nunavut
   - Ontario
   - Prince Edward Island
   - Québec
   - Saskatchewan
   - Yukon

2. What type(s) of centre do you currently work in? (Select all that apply.)
   - ☐ Academic or university-affiliated hospital or cancer centre
   - ☐ Private hospital or private treatment centre
   - ☐ Public community hospital
   - ☐ Other (please specify: _________)

3. What types of cancer do you treat? (Select all that apply.)
   - ☐ Breast
   - ☐ Endocrine and neuroendocrine
   - ☐ Gastrointestinal
   - ☐ Genitourinary
   - ☐ Gynecologic
   - ☐ Head and neck
   - ☐ Hematologic
   - ☐ Musculoskeletal
   - ☐ Neurologic
   - ☐ Ocular
   - ☐ Skin
   - ☐ Thoracic
   - ☐ Other (please specify: _____________)

4. How many years have you been in practice?
   - ☐ <5 years
   - ☐ 5-15 years
   - ☐ >15 years

5. Please indicate how the following agents are classified at your institution: (Note: drag-and-drop type question, with each regimen having to be dragged into one of 3 boxes labelled (Moderate emetic risk, High emetic risk, Not used in my practice)
   - ☐ Anthracycline/cyclophosphamide combination
   - ☐ Bendamustine
   - ☐ Busulfan
   - ☐ Carboplatin AUC ≥4
   - ☐ Carboplatin AUC <4
   - ☐ Carmustine >250 mg/$m^2$
   - ☐ Carmustine ≤250 mg/$m^2$
   - ☐ Chlorambucil
   - ☐ Cisplatin
   - ☐ Cyclophosphamide >1500 mg/$m^2$
   - ☐ Cyclophosphamide ≤1500 mg/$m^2$
   - ☐ Cytarabine >200 mg/$m^2$
   - ☐ Dacarbazine
   - ☐ Dactinomycin
   - ☐ Daunorubicin
   - ☐ Doxorubicin ≥60 mg/$m^2$
   - ☐ Doxorubicin <60 mg/$m^2$
   - ☐ Epirubicin >90 mg/$m^2$
   - ☐ Epirubicin ≤90 mg/$m^2$
   - ☐ Idarubicin
   - ☐ Ifosfamide ≥2 g/$m^2$ per dose
   - ☐ Ifosfamide <2 g/$m^2$ per dose
   - ☐ Irinotecan
   - ☐ Mechlorethamine
   - ☐ Melphalan ≥140 mg/$m^2$
   - ☐ Melphalan <140 mg/$m^2$
   - ☐ Methotrexate ≥250 mg/$m^2$
   - ☐ Mitotane
   - ☐ Oxaliplatin
   - ☐ Romidepsin
   - ☐ Sacituzumab govitecan
   - ☐ Streptozocin
   - ☐ Temozolomide
   - ☐ Trabectedin
   - ☐ Trastuzumab deruxtecan

6. For patients on the following agents, what proportion of your patients do you estimate experience with antiemetics prevention: (To enter a number from 0-100 for each category or check "Not used in my practice)

| | Anticipatory nausea/vomiting | Acute nausea | Acute vomiting | Delayed nausea | Delayed vomiting |
|---|---|---|---|---|---|
| Anthracycline/cyclophosphamide combination | | | | | |
| Carboplatin AUC ≥4 | | | | | |
| Carboplatin AUC <4 | | | | | |
| Cisplatin | | | | | |
| Sacituzumab govitecan | | | | | |
| Trastuzumab deruxtecan | | | | | |

7. What are your top 3 challenges with regards to managing CINV? (Please select 3)
   - ☐ Identifying patients on MEC at increased risk of CINV
   - ☐ Selecting appropriate antiemetic regimen
   - ☐ Access/coverage of antiemetic therapies
   - ☐ Patient compliance with antiemetic regimen
   - ☐ Anticipatory nausea/vomiting
   - ☐ Breakthrough nausea/vomiting
   - ☐ Delayed nausea/vomiting
   - ☐ Refractory nausea/vomiting
   - ☐ Other (please specify: ___________)

8. On what references for the management of CINV are your institutional protocols based? (Select all that apply.)
   - ☐ ASCO
   - ☐ CCO
   - ☐ MASCC/ESMO
   - ☐ NCCN
   - ☐ Product labelling
   - ☐ Other (please specify: _______________)

9. What prophylactic regimen do you most commonly use for patients on HEC? (To select one option in each subcategory)
   - ☐ 5-HT3 receptor antagonist
     - o Dolasetron PO
     - o Granisetron IV
     - o Granisetron PO
     - o Granisetron SC
     - o Ondansetron IV
     - o Ondansetron PO
     - o Palonosetron IV
     - o Palonosetron PO (in combination with netupitant)
     - o None
   - ☐ NK1 receptor antagonist
     - o Aprepitant IV
     - o Aprepitant PO

- Fosaprepitant IV
- Fosneptupitant IV (in combination with palonestron)
- Netupitant PO (in combination with palonestron)
- Rolapitant PO
- None

☐ Atypical antipsychotic
- Olanzapine
- None

☐ Corticosteroid
- Dexamethasone 8 mg PO x3 days
- Dexamethasone 12 mg PO or IV once
- Dexamethasone 12 mg IV on day 1, then 8 mg PO on days 2 and 3
- Other (please specify: ____________)
- None

☐ Dopamine receptor antagonist
- Metoclopramide PO
- Other (please specify: ______________)
- None

☐ Other
- Please specify: _____________
- None

10. What prophylactic regimen do you most commonly use for patients on MEC without additional risk factors for CINV?

☐ 5-HT3 receptor antagonist
- Dolasetron PO
- Granisetron IV
- Granisetron PO
- Granisetron SC
- Ondansetron IV
- Ondansetron PO
- Palonosetron IV
- Palonosetron PO (in combination with netupitant)
- None

☐ NK1 receptor antagonist
- Aprepitant IV
- Aprepitant PO
- Fosaprepitant IV
- Fosneptupitant IV (in combination with palonestron)
- Netupitant PO (in combination with palonestron)
- Rolapitant PO
- None

☐ Atypical antipsychotic
- Olanzapine
- None

☐ Corticosteroid
- Dexamethasone 8 mg PO x3 days

        - Dexamethasone 12 mg PO or IV once
        - Dexamethasone 12 mg IV on day 1, then 8 mg PO on days 2 and 3
        - Other (please specify: ____________)
        - None
    - Dopamine receptor antagonist
        - Metoclopramide PO
        - Other (please specify: ______________)
        - None
    - Other
        - Please specify: _____________
        - None

11. What prophylactic regimen do you most commonly use for patients on MEC with additional risk factors for CINV?
    - 5-HT3 receptor antagonist
        - Dolasetron PO
        - Granisetron IV
        - Granisetron PO
        - Granisetron SC
        - Ondansetron IV
        - Ondansetron PO
        - Palonosetron IV
        - Palonosetron PO (in combination with netupitant)
        - None
    - NK1 receptor antagonist
        - Aprepitant IV
        - Aprepitant PO
        - Fosaprepitant IV
        - Fosneptupitant IV (in combination with palonestron)
        - Netupitant PO (in combination with palonestron)
        - Rolapitant PO
        - None
    - Atypical antipsychotic
        - Olanzapine
        - None
    - Corticosteroid
        - Dexamethasone 8 mg PO x3 days
        - Dexamethasone 12 mg PO or IV once
        - Dexamethasone 12 mg IV on day 1, then 8 mg PO on days 2 and 3
        - Other (please specify: ____________)
        - None
    - Dopamine receptor antagonist
        - Metoclopramide PO
        - Other (please specify: ______________)
        - None
    - Other
        - Please specify: _____________

- None

12. What risk factors do you consider when determining whether to initiate an NK-1 RA in patients on MEC? (Select all that apply.)
    - ☐ Age
    - ☐ Gender
    - ☐ BMI
    - ☐ Performance status
    - ☐ Anticancer regimen
    - ☐ Number of lines of prior treatment
    - ☐ Anticipatory nausea/vomiting with prior therapy
    - ☐ CINV with prior therapy
    - ☐ History of low alcohol intake
    - ☐ History of morning sickness
    - ☐ Comorbid anxiety disorder
    - ☐ Comorbid GERD
    - ☐ Concurrent radiation
    - ☐ Concurrent CYP-inducing medication
    - ☐ Other (please specify: ____________)

13. At your institution, how are patients assessed to determine if they have experienced any nausea and/or vomiting? (Select all that apply.)
    - ☐ In-person by:
        - Nurse
        - Oncologist
        - Pharmacist
    - ☐ Telephone by:
        - Nurse
        - Oncologist
        - Pharmacist
    - ☐ Patient encouraged to call if they experience nausea and/or vomiting
    - ☐ Other (please specify: _________)

14. At your institution, at what timepoints are patients assessed to determine if they have experienced any nausea and/or vomiting? (Select all that apply.)
    - ☐ Prior to administration
    - ☐ Immediately after administration
    - ☐ 1-2 days after administration
    - ☐ 3-4 days after administration
    - ☐ 5-6 days after administration
    - ☐ 1 week after administration
    - ☐ 2 weeks after administration
    - ☐ 3 weeks after administration
    - ☐ Patient encouraged to call if they experience nausea and/or vomiting
    - ☐ Other (please specify: ___________)

## PATIENT PROFILE

To select: 10 adults who have been, or will be, treated with a high or moderately emetogenic anticancer regimen.

1. Age:
   - ☐ 18-29 years
   - ☐ 30-39 years
   - ☐ 40-49 years
   - ☐ 50-59 years
   - ☐ 60-69 years
   - ☐ 70-79 years
   - ☐ ≥80 years

2. Gender:
   - ☐ Female
   - ☐ Male
   - ☐ Other

3. Eastern Cooperative Oncology Group (ECOG) performance status:
   - ☐ 0
   - ☐ 1
   - ☐ 2
   - ☐ 3
   - ☐ 4

4. Karnofsky performance status: ___ (drop-down list of numbers 0-100)

5. Type of coverage (private/public):
   - ☐ Private
   - ☐ Public
   - ☐ Other (please specify: ___________)

6. Type of cancer:
   - ☐ Breast
   - ☐ Endocrine and neuroendocrine
   - ☐ Gastrointestinal
   - ☐ Genitourinary
   - ☐ Gynecologic
   - ☐ Head and neck
   - ☐ Hematologic
   - ☐ Musculoskeletal
   - ☐ Neurologic
   - ☐ Ocular
   - ☐ Skin
   - ☐ Thoracic
   - ☐ Other (please specify: ______________)

7. Stage of cancer:
   - ☐ I
   - ☐ II
   - ☐ II
   - ☐ IV

8. Presence of brain metastases and/or carcinomatous meningitis:
   - ☐ Yes
   - ☐ No

9. Presence of peritoneal carcinomatosis with or without bowel obstruction:
   - ☐ Yes
   - ☐ No

10. Treatment setting:
    - ☐ Neoadjuvant
    - ☐ Adjuvant
    - ☐ Maintenance
    - ☐ Metastatic

11. Current or planned treatment regimen:
    - ☐ Moderate emetic risk
    - ☐ High emetic risk

12. Number of treatment cycles received: ___ (drop-down list of numbers 0-99)

13. Is there ongoing radiation?
    - ☐ Yes
    - ☐ No

14. Is there other ongoing anticancer therapy? (Select all that apply.)
    - ☐ Immunotherapy
    - ☐ Targeted therapy
    - ☐ Other (please specify: ________)
    - ☐ None

15. Potential risk factors for CINV: (Select all that apply.)
    - ☐ Anticipatory nausea/vomiting with prior therapy
    - ☐ CINV with prior therapy
    - ☐ Delayed nausea/vomiting with prior therapy
    - ☐ Anticipatory nausea/vomiting in prior cycles
    - ☐ CINV with prior cycles
    - ☐ Delayed nausea/vomiting with prior cycles
    - ☐ BMI <27.5 $kg/m^2$
    - ☐ History of low alcohol intake

- ☐ History of morning sickness
- ☐ Other (please specify: __________)

16. Comorbidities: (Select all that apply.)
    - ☐ Anxiety disorder
    - ☐ Arrhythmia
    - ☐ Cognitive impairment
    - ☐ Diabetes
    - ☐ Electrolyte or metabolic disorder (e.g., hypercalcemia, hyponatremia)
    - ☐ GERD
    - ☐ Kidney disease
    - ☐ Liver disease
    - ☐ QTc prolongation
    - ☐ Severe pain
    - ☐ Other relevant comorbidity (please specify: ___________)
    - ☐ None of the above

17. Concomitant medications:
    - ☐ Antibiotic
    - ☐ Antidepressant
    - ☐ Antipsychotic
    - ☐ CYP inducer
    - ☐ Opioid
    - ☐ Other relevant medication (please specify: ___________)
    - ☐ None of the above

18. What topics were discussed with the patient regarding CINV management? (Select all that apply.)
    - ☐ Risk of CINV with their anticancer regimen
    - ☐ Personal risk factors for CINV
    - ☐ Anticipatory nausea/vomiting
    - ☐ Breakthrough nausea/vomiting
    - ☐ Delayed nausea/vomiting
    - ☐ Non-pharmacological strategies for CINV management
    - ☐ Pharmacologic strategies for CINV management
    - ☐ Patient preferences
    - ☐ Other (please specify: ________)
    - ☐ None of the above

19. Was a prophylactic antiemetic regimen selected for this patient?
    - ☐ Yes
    - ☐ No

*Following question to appear only if "No" selected*

20. Why was a prophylactic antiemetic regimen not selected? (Select all that apply.)
    - ☐ Lack of access/coverage

- ☐ Patient deemed at lower risk for CINV
- ☐ Regimen was offered but refused by patient
- ☐ Reactive antinausea medication provided instead
- ☐ Other (please specify: ___________)

*Following questions to appear only if "Yes" selected*

21. Prior to which treatment cycle was antiemetic regimen started? ___ (drop-down list of numbers 1-99)

22. What regimen was selected: (To select one option in each subcategory)
    - ☐ 5-HT3 receptor antagonist
        - Dolasetron PO
        - Granisetron IV
        - Granisetron PO
        - Granisetron SC
        - Ondansetron IV
        - Ondansetron PO
        - Palonosetron IV
        - Palonosetron PO (in combination with netupitant)
        - None
    - ☐ NK1 receptor antagonist
        - Aprepitant IV
        - Aprepitant PO
        - Fosaprepitant IV
        - Fosneptupitant IV (in combination with palonestron)
        - Netupitant PO (in combination with palonestron)
        - Rolapitant PO
        - None
    - ☐ Atypical antipsychotic
        - Olanzapine as prophylaxis
        - Olanzapine for breakthrough CINV
        - Other (please specify: _________)
        - None
    - ☐ Corticosteroid
        - Dexamethasone 8 mg PO x3 days
        - Dexamethasone 12 mg PO or IV once
        - Dexamethasone 12 mg IV on day 1, then 8 mg PO on days 2 and 3
        - Other (please specify: ____________)
        - None
    - ☐ Dopamine receptor antagonist
        - Metoclopramide PO
        - Other (please specify: ______________)
        - None
    - ☐ Other
        - Please specify: _____________
        - None
